# Inhibiting BDNF Signaling Upregulates Hippocampal H3K9me3 in a Manner Dependent On *In Vitro* Aging and Oxidative Stress

**DOI:** 10.3389/fragi.2022.796087

**Published:** 2022-04-28

**Authors:** Andra Ionescu-Tucker, Liqi Tong, Nicole C. Berchtold, Carl W. Cotman

**Affiliations:** ^1^ Department of Neurobiology and Behavior, University of California, Irvine, Irvine, CA, United States; ^2^ Institute for Memory Impairments and Neurological Disorders, University of California, Irvine, Irvine, CA, United States

**Keywords:** BDNF, H3K9me3, epigenetics, hippocampus, oxidative stress

## Abstract

Histone modifications are key contributors to the cognitive decline that occurs in aging and Alzheimer’s disease. Our lab has previously shown that elevated H3K9me3 in aged mice is correlated with synaptic loss, cognitive impairment and a reduction in brain derived neurotrophic factor (BDNF). However, the mechanism of H3K9me3 regulation remains poorly understood. In this study, we investigated the role of age-associated stressors on H3K9me3 regulation and examined if changes in H3K9me3 were age dependent. We used cultured hippocampal neurons at 6, 12, and 21 days *in vitro* (DIV) to examine the effect of different stressors on H3K9me3 across neuron ages. We found that the oxidative stressor hydrogen peroxide (H2O2) does not induce H3K9me3 in 12 DIV neurons. Inhibiting BDNF signaling *via* TrkB-Fc elevated H3K9me3 in 12 and 21 DIV neurons compared to 6 DIV neurons. Antioxidant treatment prevented H3K9me3 elevation in 12 DIV neurons treated with TrkB-Fc and H2O2. H2O2 elevated the epigenetic regulator SIRT1 in 6 DIV neurons but did not increase H3K9me3 levels. Our findings demonstrate that inhibiting BDNF signaling elevates hippocampal H3K9me3 in a manner dependent on in vitro age and oxidative stress.

## Introduction

Histone modifications are emerging as a key contributor to the cognitive decline that occurs in aging and neurodegeneration (Lardenoije et al., 2015). Recent studies suggest that cognitive functions in aging are constrained by repressive histone methylation and investigating these epigenetic blockades may clarify the mechanism of age-related cognitive decline (Gupta et al., 2010; Day and Sweatt, 2011). A highly regulated site is histone 3, lysine 9 (H3K9) which is associated with gene silencing when di or tri-methylated (Peters et al., 2003; Stewart et al., 2005; Barter and Foster, 2018). H3K9 is trimethylated (me3) by the histone methyltransferase SUV39H1, and H3K9me3 has a critical role in aging, including vascular inflammation and diabetes (Sedivy et al., 2008; Villeneuve et al., 2010). We thus identified H3K9me3 as a potential target for epigenetic regulation of age-associated cognitive decline.

Previous work by our lab has shown that H3K9me3 increases in the mouse hippocampus with age. To investigate if H3K9me3 was associated with age-related cognitive decline, we reduced H3K9me3 in aged mice using a selective SUV39H1 inhibitor (ETP69), which improved performance in object location memory (OLM) and fear conditioning tasks. ETP69 further increased the expression of brain derived neurotrophic factor (BDNF) while decreasing H3K9me3 at BDNF promoter 1 (Snigdha et al., 2016). This indicates that H3K9me3 regulates the expression of a neurotrophic factor that is critical for memory, neuron survival and brain plasticity (Berchtold et al., 2002; Cotman and Berchtold, 2002). Our work indicates an important role for H3K9me3 in the regulation of brain health and demonstrates a clear association between hippocampal H3K9me3 and age. However, the mechanism underlying H3K9me3 elevation in the aging hippocampus has yet to be explored.

Studies suggest that oxidative stress, which is elevated with age, may lead to H3K9me3 elevation (Harman, 1992; Butterfield and Halliwell, 2019). Hydrogen peroxide (H2O2) increased H3K9me3 in human embryonic kidney cells by upregulating SIRT1, a sirtuin that promotes protective responses to oxidative stress. SIRT1 stabilizes the histone methyltransferase SUV39H1, and SIRT1’s upregulation activates SUV39H1 and increases H3K9me3 levels (Vaquero et al., 2007). A similar study found that H2O2 elevates SIRT1, SUV39H1 and H3K9me3 in mouse embryonic fibroblasts, and that SIRT1 knock-out reduces SUV39H1 (Bosch-Presegué et al., 2011). Conversely, oxidation in rat neonatal ventricular monocytes upregulates SUV39H1 while downregulating SIRT1, as SUV39H1 catalyzes repressive H3K9 trimethylation on the SIRT1 promoter (Yang et al., 2017). Regulation of SIRT1 may thus be tissue dependent, and SIRT1’s role in hippocampal H3K9 trimethylation has not been defined.

In this study, we investigated H3K9me3’s regulation in the hippocampus by determining which age-associated stressors lead to its elevation. We examined if oxidative stress upregulated H3K9me3 and found that H2O2 did not significantly elevate H3K9me3 in 12 DIV hippocampal neurons. We next examined if inhibiting BDNF signaling increased levels of H3K9me3, as BDNF is reduced in the aged brain and degrades SUV39H1 in cortical neurons (Sen and Snyder, 2011; Erickson et al., 2012). We found that both inhibiting BDNF signaling (TrkB-Fc) and a combination of TrkB-Fc and H2O2 significantly elevated H3K9me3 in 12 DIV hippocampal neurons. We then examined if H3K9me3’s susceptibility to stressors was dependent on neuron maturation by examining H3K9me3 levels in 6 and 21 DIV hippocampal neurons in addition to 12 DIV cultures. We further investigated if antioxidant supplementation prevented an oxidative stress-induced increase in H3K9me3. Lastly, we determined how SIRT1 levels correlated with H3K9me3 elevation. We found that hippocampal neurons were more susceptible to stress-induced elevations in H3K9me3 as they matured and that antioxidant treatment prevents H3K9me3 elevation in 12 DIV neurons treated with TrkB-Fc and H2O2. SIRT1 levels were elevated in 6 DIV neurons treated with H2O2 and did not correlate with H3K9me3 levels, suggesting that SIRT1 does not mediate H3K9 trimethylation in hippocampal neurons. Overall, this study has demonstrated that inhibiting BDNF signaling promotes hippocampal H3K9 trimethylation in a manner dependent on *in vitro* age and oxidative stress.

## Results

### Hydrogen Peroxide Does Not Induce H3K9me3 in 12 DIV Hippocampal Neurons

**FIGURE 1:**
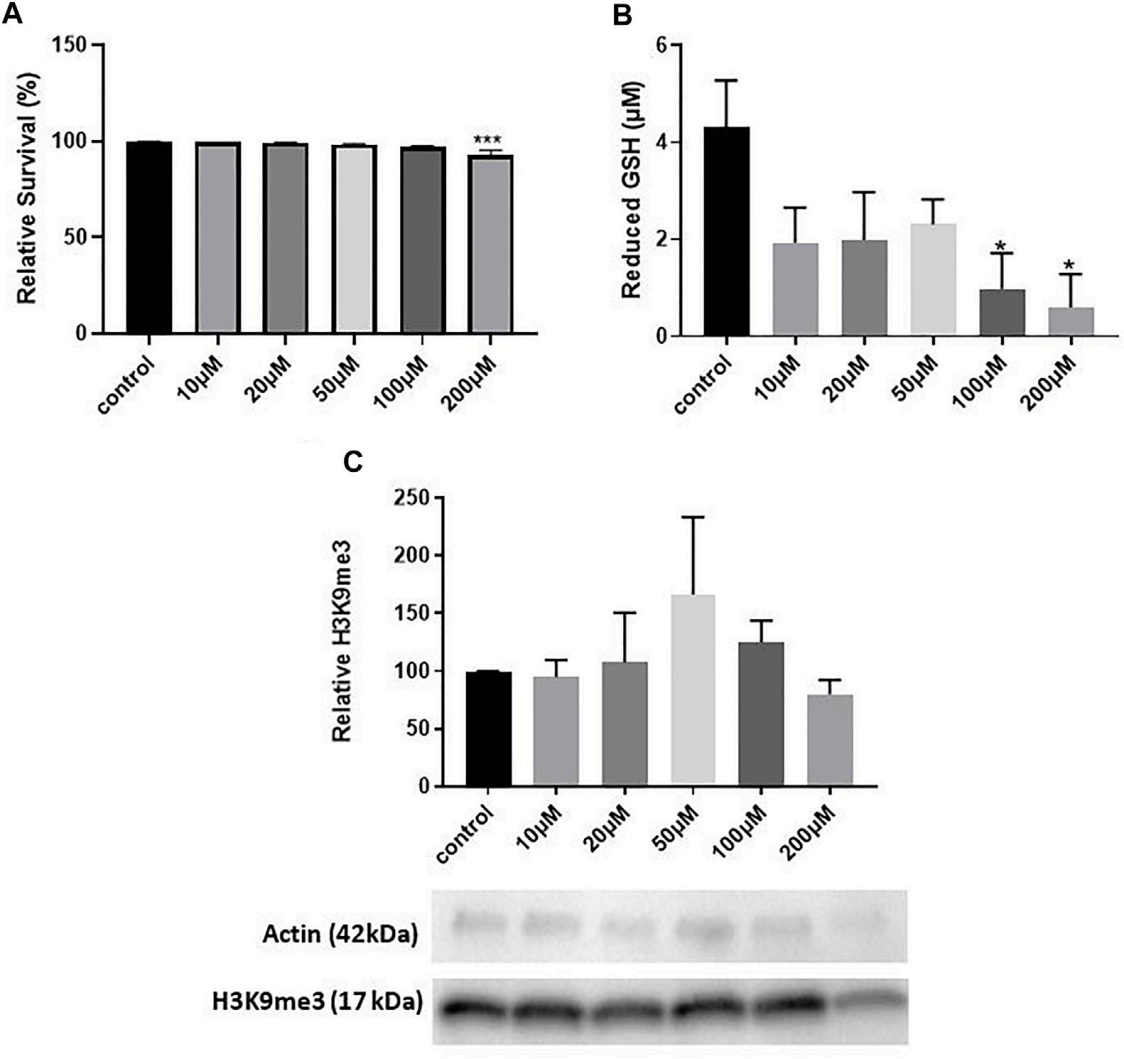
The Age-Associated Stressor H2O2 Does Not Induce Repressive Histone Methylation in 12 DIV Hippocampal Neurons. **(A)** LDH assay quantification of toxicity in 12 DIV hippocampal neuron cultures treated with 10–200 μM H2O2. One-way ANOVA, ***p = 0.0025, *n* = 4. Dunnet’s multiple comparisons test, ***p = 0.0010 control vs. 200 μM. **(B)** Glutathione assay for 12 DIV hippocampal neuron cultures treated with 10–200 μM H2O2. One way ANOVA, *p = 0.041, *n* = 4. Dunnett’s multiple comparisons test,*p = 0.02 100 μM vs. control, *p = 0.012 200 μM vs. control. **(C)** Quantification of H3K9me3 in 12 DIV hippocampal neuron cultures. One way ANOVA, *n* = 4, *p* = 0.68. All cultures were treated with H2O2 for 5 min and harvested 8 h later.

We first conducted a dose-response study to determine a concentration of hydrogen peroxide that significantly elevated oxidative stress. We used 12 DIV hippocampal neuron cultures and treatment with varying concentrations of H2O2 previously shown to induce oxidative stress by our lab (Whittemore et al., 1995). We treated cultures with H2O2 for 5 min, replaced the media and harvested cultures 8 h later, a treatment paradigm that induces epigenetic changes in mouse hippocampal neuron cultures (Gräff et al., 2012). LDH assay quantification indicated a significant decrease in survival with 200 μM H2O2 (One way ANOVA, *p = 0.0025; Tukey’s post hoc test, ***p = 0.0010 relative to control) which corresponds to approximately a 7% decrease in neuron survival (Figure 1A). 100 and 200 μM H2O2 decreased the antioxidant reduced glutathione (GSH) (One way ANOVA, *p = 0.041; Dunnett’s post hoc test, *p = 0.02 100 μM relative to control, **p = 0.012 200 μM relative to control), indicating that these concentrations of H2O2 significantly increase oxidative stress (Figure 1B). However, H2O2 did not significantly change H3K9me3 levels (One way ANOVA, *p* = 0.68) (Figure 1C). While we optimized two concentrations of hydrogen peroxide that induced oxidative stress (100 and 200 μM), neither of these concentrations were sufficient to elicit a change in hippocampal H3K9me3. We concluded that hydrogen peroxide was insufficient to elicit a change in H3K9me3 in 12 DIV neuron cultures.

### Inhibiting BDNF Signaling Significantly Elevates H3K9me3 in 12 DIV Hippocampal Neurons

**FIGURE 2:**
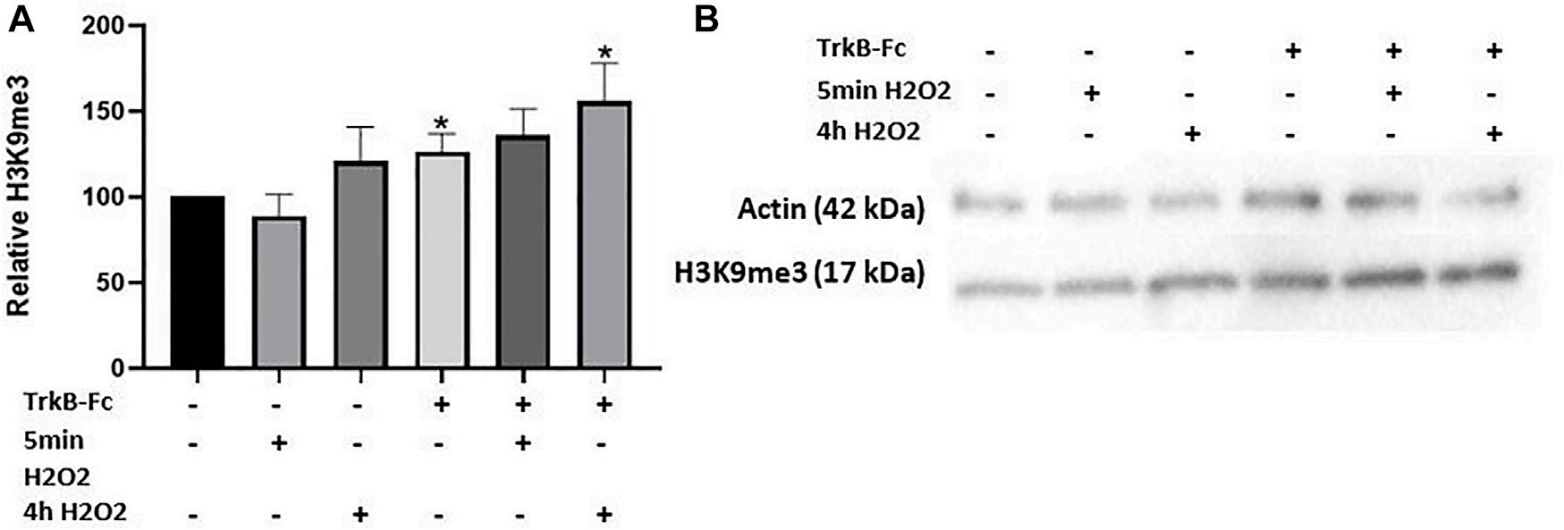
TrkB-Fc and 4h H2O2 Significantly Elevate H3K9me3 in 12 DIV Neuron Cultures. **(A)** H3K9me3 western blot analysis of 12 DIV hippocampal neuron cultures threated with 200 μM H2O2 for 5 min 8 h before harvest or 4 h, 1 μg/ml TrkB-Fc for 24 h. Welch’s ANOVA, *p* = 0.033, *n* = 9 for control, TrkB-Fc, 4 h H2O2 and TrkB-Fc + 4 h H2O2; *n* = 4 for 5 min H2O2 and TrkB-Fc + 5 min H2O2. Dunnett’s T3 multiple comparisons test,*p = 0.033 control vs. TrkB-Fc + 4 h H2O2, *p = 0.037 control vs. TrkB-Fc. **(B)** Representative western blot images for H3K9me3 and actin (loading control).

We then investigated if a combination of oxidative stress and impaired BDNF signaling could change H3K9me3 levels, as reduced BDNF is another age-associated stressor (Erickson, 2012). We used a concentration of a BDNF receptor inhibitor (TrkB-Fc) that had previously been optimized in our lab for cell culture (24 h, 1 μg/ml; data not shown). 200 μM H2O2 was used as this concentration produced the greatest level of oxidative stress considering its small effect on neuron survival (Figures 1A,B). We further tested if a longer H2O2 treatment time (4 h) could change H3K9me3 levels as a brief treatment period had no effect. H2O2 loses its reactivity quickly in dilute solutions, so we did not expect a significant change in neuron survival (Whittemore et al., 1995). We found that neuron survival was not significantly affected as measured by an LDH assay (Supplementary Figure S1; One way ANOVA, *p = 0.039, *n* = 4). Both TrkB-Fc alone and in combination with 4 h H2O2 treatment induced a significant increase in H3K9me3 (Welch’s ANOVA, *p* = 0.033, *n* = 9; Dunnett’s post hoc test,*p = 0.033 control vs. TrkB-Fc + 4 h H2O2, *p = 0.037 control vs. TrkB-Fc) (Figure 2A). Our findings indicate that inhibiting BDNF signaling significantly elevates H3K9me3 in 12 DIV hippocampal neurons.

### Hippocampal Neurons Become More Susceptible to Stressor Induced H3K9me3 With Neuronal Maturation

To determine if stress induced changes in H3K9me3 were dependent on neuronal maturation, we compared the effects of inhibiting BDNF signaling and oxidative stress across neuron ages (6, 12 and 21 DIV neurons). A previous study used short-term (4-8 DIV) and long-term cultures (15–21 DIV) to resemble “young” and “aged” neurons in the rat hippocampus, as rat hippocampal neurons have markers of aging after several weeks in culture, including increased oxidative damage (Calvo-Rodríguez et al., 2016). We thus used different stages of in vitro neuronal maturation as a model for neuronal aging. As 5 min of H2O2 treatment did not change H3K9me3 in 12 DIV neurons (Figure 2A), we did not use this treatment condition in later experiments.

**FIGURE 3:**
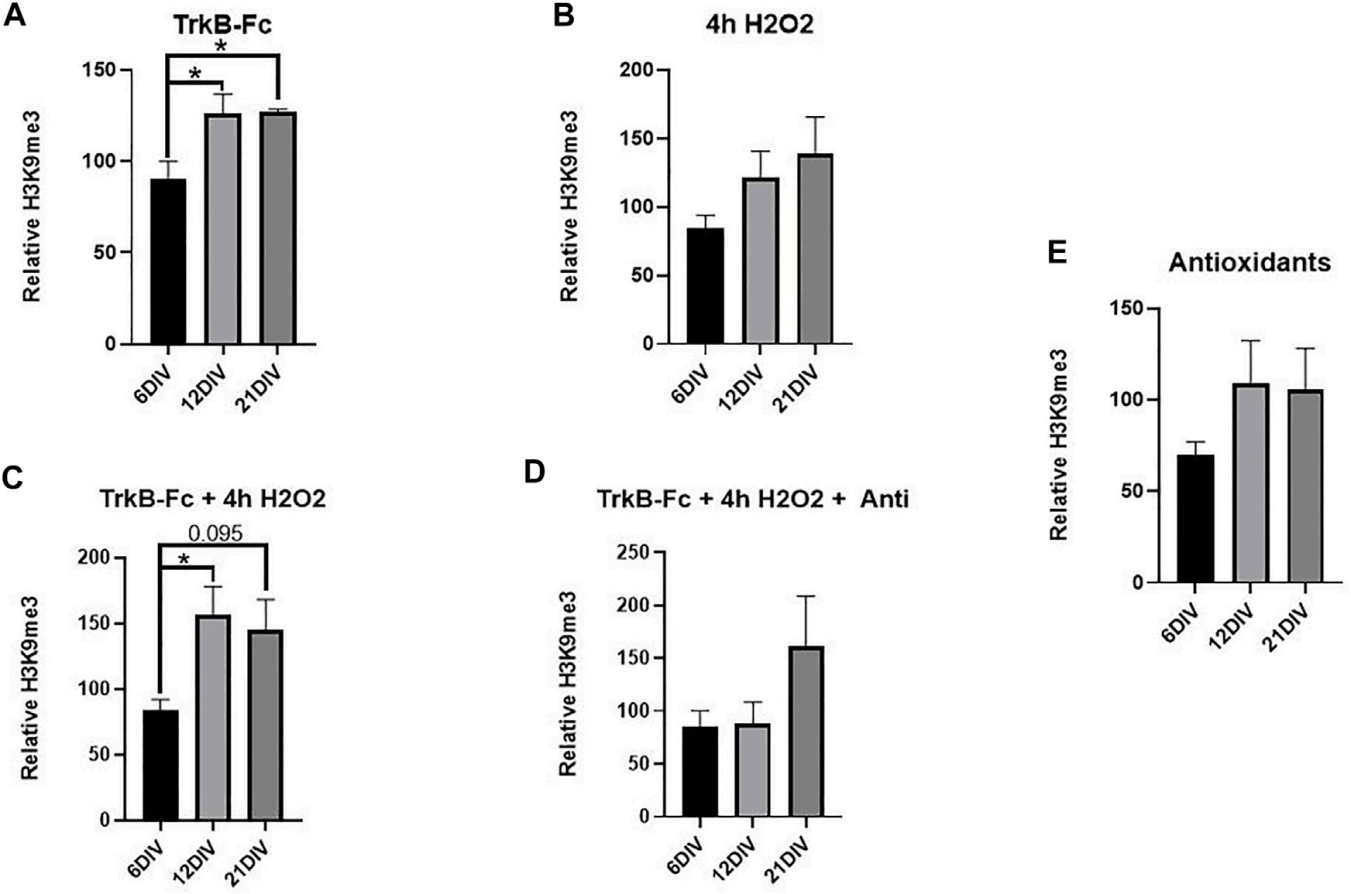
TrkB-Fc Significantly Elevates H3K9me3 in 12 and 21 DIV Hippocampal Neuron Cultures. 12 and 21 DIV were compared to 6 DIV. Relative H3K9me3 levels are normalized to untreated controls for each culture age. **(A)** H3K9me3 western blot analysis of hippocampal neuron cultures treated with 1 μg/ml TrkB = TrkB-Fc for 24 h. Welch’s ANOVA, *p = 0.015; *n* = 7 for 6 DIV, *n* = 9 for 12 DIV, *n* = 5 for 21 DIV. Dunnet’s T3 multiple comparisons test, *p = 0.018 6 DIV vs. 21 DIV, *p = 0.048 6 DIV vs. 12 DIV. **(B)** H3K9me3 western blot analysis of hippocampal neuron cultures treated with 200 μM H2O2 for 4 h. Welch’s ANOVA, *p* = 0.12; *n* = 7 for 6 DIV, *n* = 9 for 12 DIV, *n* = 5 for 21 DIV. **(C)** H3K9me3 western blot analysis of hippocampal neuron cultures treated with 1 μg/ml TrkB-Fc for 24 h and 200 μM H2O2 for 4 h. Welch’s ANOVA, *p = 0.018, *n* = 7 for 6 DIV, *n* = 9 for 12 DIV, *n* = 5 for 21 DIV. Dunnett’s T3 multiple comparisons test, *p = 0.022 6 DIV vs. 12 DIV, *p* = 0.095 6DIV vs. 21 DIV. **(D)** H3K9me3 western blot analysis of hippocampal neurons treated with 1 μg/ml TrkB-Fc for 24 h, 200 μM H2O2 for 4 h and antioxidants for 24 h. Welch’s ANOVA, *p* = 0.44; *n* = 7 for 6 div, *n* = 4 for 12 DIV and *n* = 3 for 21 DIV. **(E)** H3K9me3 western blot analysis of hippocampal neurons treated with antioxidants for 24 h. Welch’s ANOVA, *p* = 0.27; *n* = 7 for 6 DIV, *n* = 4 for 12 DIV and *n* = 3 for 21 DIV.

Neuron survival was not affected by TrkB-Fc, H2O2 or antioxidants at any culture maturation stage (Supplementary Figure S1). TrkB-Fc treatment significantly increased H3K9me3 in 21 DIV neuron cultures and 12 DIV neuron cultures compared to 6 DIV cultures (Figure 3A, Welch’s ANOVA, *p = 0.018 6 DIV vs. 21 DIV, *p = 0.048 6 DIV vs. 12 DIV). TrkB-Fc in combination with 4 h H2O2 increased H3K9me3 in 12 DIV cultures relative to 6 DIV cultures (Figure 3C, Welch’s ANOVA, *p = 0.032 6 DIV vs. 12 DIV), while H3K9me3 levels in 21 DIV cultures failed to reach statistical significance (*p* = 0.095). Consistent with our finding that H2O2 does not affect H3K9me3 in 12 DIV neurons (Figure 1C), 4h of H2O2 treatment did not affect H3K9me3 levels at any culture age (Figure 3B, Welch’s ANOVA, *p = 0.12). These findings indicate that while inhibiting BDNF signaling can induce H3K9me3 in 21 DIV hippocampal neurons, additional oxidative stress may prevent regulation of H3K9me3 by BDNF. The mechanism of H3K9me3 regulation is thus highly dependent on neuronal maturation, and 12 DIV neurons display the greatest elevation in H3K9me3 when treated with exogenous stressors.

### Antioxidants Prevent H3K9me3 Upregulation in 12 DIV Hippocampal Neurons

We examined if antioxidant treatment could change H3K9me3 levels in hippocampal neurons treated with TrkB-Fc and H2O2. Antioxidant treated cultures were fed with B27 media as opposed to standard treatment media (B27 without antioxidants) 24 h before harvesting. Antioxidant treated cultures were thus supplemented with a proprietary mix of vitamin E, vitamin E acetate, superoxide dismutase, catalase, and glutathione (Fisher 17504044).

We hypothesized that antioxidant treatment would ameliorate the oxidative stress produced by H2O2, elevating H3K9me3 in 21 DIV neurons in a manner similar to TrkB-Fc alone (Figure 3A). There was no significant difference in H3K9me3 between 6, 12, and 21 DIV neurons treated with TrkB-Fc, H2O2 and antioxidants. This suggests that antioxidant treatment prevented a significant H3K9me3 elevation in 12 DIV neurons treated with TrkB-Fc and H2O2 (Figures 3C,D). Antioxidant treatment had no effect on H3K9me3 in unstressed hippocampal neurons (Figure 3E).

### SIRT1 Is Elevated in 6 DIV Hippocampal Neurons Treated With H2O2

**FIGURE 4:**
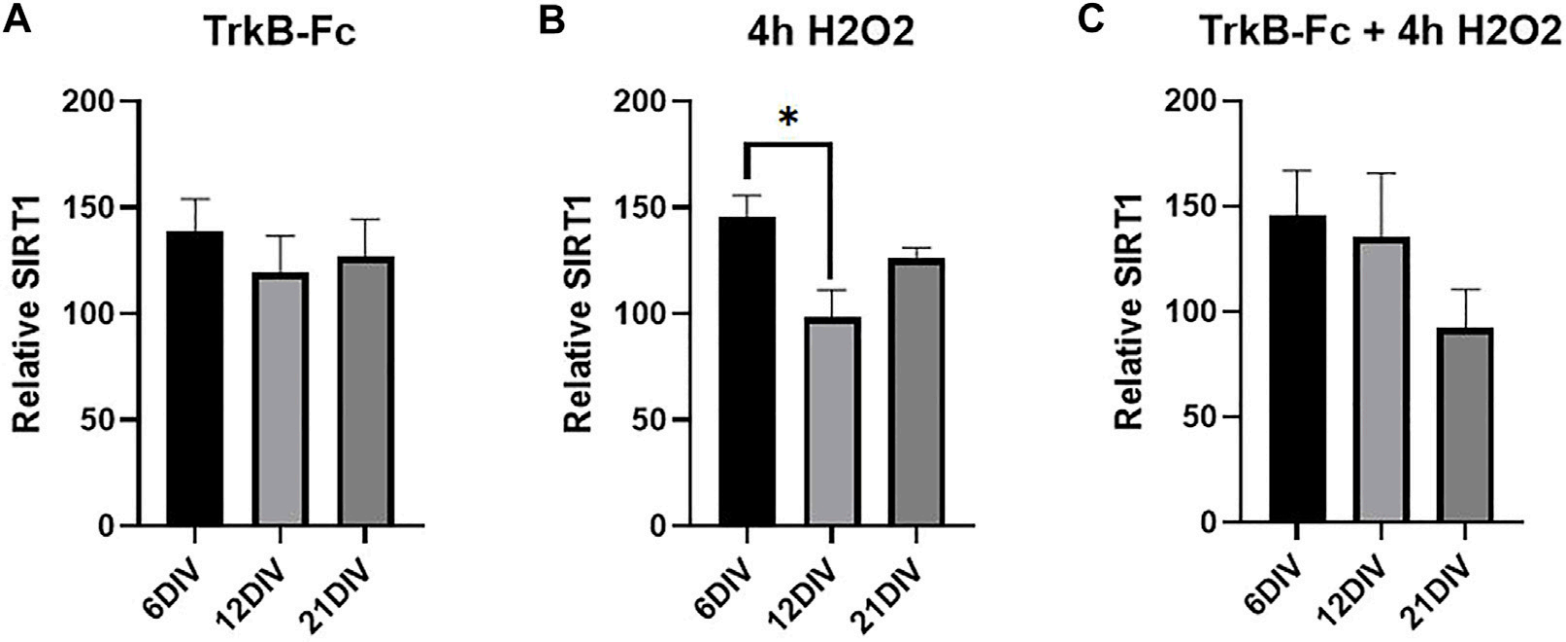
SIRT1 is Elevated in 6 DIV Neurons Treated with Hydrogen Peroxide. **(A)** SIRT1 western blot analysis of hippocampal neurons treated with 1 μg/ml TrkB-Fc for 24 h. Welch’s ANOVA, *p* = 0.72. **(B)** SIRT1 western blot analysis of hippocampal neurons treated with 200 μM H2O2 for 4 h. Welch’s ANOVA *p = 0.011; Dunnett’s T3 multiple comparisons test, *p = 0.037 6 DIV vs. 12 DIV. **(C)** SIRT1 western blot analysis of hippocampal neurons treated with 1 μg/ml TrkB-Fc for 24 h and 200 μM H2O2 for 4 h. Welch’s ANOVA, *p* = 0.21. N = 5 for 6 DIV, *n* = 9 for 12 DIV, *n* = 4 for 21 DIV.

Studies in non-neuronal tissues indicate that SIRT1 mediates oxidative stress-induced epigenetic changes (Vaquero et al., 2007; Bosch-Presegué et al., 2011; Yang et al., 2017). We measured SIRT1 levels in our stressed cultures to determine if the same treatments that increased H3K9me3 also elevated SIRT1 levels. SIRT1 was significantly increased in 6 DIV neurons compared to 12 DIV neurons treated with H2O2 (Figure 4B, Welch’s ANOVA, *p = 0.037). Similarly, there was no change in SIRT1 levels in antioxidant treated cultures (Supplementary Figure S3). H2O2 increased SIRT1 but had no effect on H3K9me3 (Figure 3B), and there was no clear relationship between the two markers in hippocampal neurons. While hydrogen peroxide does elevate SIRT1 in 6 DIV neurons, SIRT1 upregulation does not lead to H3K9 trimethylation in hippocampal neuron cultures.

## Discussion

In this study we found that inhibiting BDNF signaling elevates H3K9me3 in 12 and 21 DIV, but not 6 DIV, hippocampal neurons. While H2O2 alone did not affect H3K9me3 levels at any neuron age, antioxidant treatment prevented H3K9me3 elevation in 12 DIV neurons treated with TrkB-Fc and H2O2. Surprisingly, there was no change in SIRT1 in cultures with elevated H3K9 trimethylation. Our findings suggest that neurotrophic deprivation elevates H3K9me3 in a manner depending on *in vitro* age and oxidative stress.

### Oxidative Stress Alone Does Not Regulate Hippocampal H3K9me3

Previous studies have shown that H2O2 upregulates H3K9me3 in non-neuronal tissues (Vaquero et al., 2007; Bosch-Presegué et al., 2011; Yang et al., 2017). However, H2O2 alone did not elevate H3K9me3 in our hippocampal neuron cultures. Mechanisms of epigenetic regulation may differ in neurons since they have greater levels of endogenous oxidative stress than other cell types. Neurons are post-mitotic cells that cannot be replaced and accumulate oxidative and mitochondrial damage as they age (Grimm and Eckert, 2017). Out of all brain regions, oxidative stress is highest in the hippocampus, partly due to high levels of reactive oxygen species (ROS) in the CA1 (Wang and Michaelis, 2010). BDNF protects neurons from excess oxidative stress by increasing levels of superoxide dismutase (SOD) and glutathione reductase (GRX) in cultured rat hippocampal neurons (Mattson et al., 1995; Chen et al., 2017). A study in rat PC12 cells found that sublethal levels of H2O2 increase BDNF expression, suggesting that H2O2 might further increase levels of BDNF in our cultures (Ogura et al., 2014). Although H2O2 did significantly elevate oxidative stress in hippocampal neuron cultures, this stress is likely reduced by BDNF, preventing H2O2 alone from eliciting an effect on H3K9me3 (Figures 1B,C). In sum, BDNF protects against oxidative stressors like H2O2 and may even be induced by high levels of oxidative stress. Our findings show that H3K9me3 levels are only elevated once BDNF signaling is inhibited.

### Inhibiting BDNF Signaling Elevates H3K9me3 in an Age-Dependent Manner

Inhibition of BDNF signaling (TrkB-Fc) elevated H3K9me3 in 12 DIV neurons by itself and in combination with H2O2 (Figure 2A). This is consistent with a study showing that BDNF signaling leads to SUV39H1 degradation in cortical neurons (Sen and Snyder, 2011). We hypothesized that hippocampal neurons would become more susceptible to stress induced epigenetic changes with maturation due to the accumulation of oxidative damage (Wang and Michaelis, 2010). We thus expected that H3K9me3 would not be elevated in 6 DIV neurons by TrkB-Fc or H2O2, while 21 DIV neurons would show a similar or greater increase in H3K9me3 with TrkB-Fc and H2O2. As predicted, stressors did not increase H3K9me3 in 6 DIV neurons, and TrkB-Fc increased H3K9me3 in 21 DIV neurons (Figure 3A). However, the combination of TrkB-Fc and H2O2 did not significantly increase H3K9me3 in 21 DIV neurons, although there was an increasing trend (Figure 3C). Regulation of H3K9me3 may be impaired in 21 DIV neurons by high levels of oxidative stress, such that excess stress does not stimulate H3K9 trimethylation. The increased sensitivity of 21 DIV cultures also made them more challenging to produce than less stressed 6 and 12 DIV neurons. A recent study found that although hippocampal H3K9me3 was elevated in both adult and aged mice, the greatest elevation was in adult mice (Kushwaha and Thakur, 2020). This is consistent with our prior research since we did not examine the adult mouse population and found that H3K9me3 was elevated in aged mice in comparison to young mice (Snigdha et al., 2016). While neurons are more susceptible to epigenetic changes as they age, the regulation of H3K9me3 may be impaired in 21 DIV neurons due to higher levels of oxidative stress. Further research is needed to determine how the mechanism of H3K9me3 regulation changes with increased neuronal maturation.

### Antioxidants Prevent H3K9me3 Elevation Caused By Inhibiting BDNF Signaling

Although antioxidants had no effect on H3K9me3 in unstressed cultures, antioxidant treatment prevented H3K9me3 elevation in 12 DIV neurons treated with TrkB-Fc and H2O2 (Figure 3D). This shows that antioxidants by themselves are not sufficient to reduce H3K9me3. Indeed, H3K9me3 appears to be relatively stable due to its critical role in the maintenance of heterochromatin stability and double strand break repair (Ayrapetov et al., 2014). While antioxidants cannot reduce H3K9me3 from baseline levels, they can prevent an elevation in H3K9me3 in stressed cultures where BDNF is inhibited. Impairment of BDNF signaling reduces antioxidants and antioxidant supplementation prevents H3K9me3 from increasing, suggesting that the oxidative stress caused by inhibiting BDNF signaling elevates H3K9me3 (Mattson et al., 1995; Chen et al., 2017). Oxidative stress is responsible for H3K9me3 elevation in neurons as in other tissue types, but a critical source of protective antioxidants (BDNF) must be inhibited to reach a ROS threshold that can increase H3K9me3.

### H2O2 Elevates SIRT1 But Not H3K9me3

Our findings suggest that inhibiting BDNF signaling upregulates H3K9me3 *via* an oxidative stress dependent mechanism. Various studies have shown that oxidative stress (specifically H2O2) elevates SIRT1 and in turn H3K9me3 in non-neuronal cells (Vaquero et al., 2007; Bosque-Presegué et al., 2011; Yang et al., 2017). However, H202 elevated SIRT1 but did not affect H3K9me3 levels in hippocampal neuron cultures (Figure 3B) and (Figure 4B). SIRT1 was only significantly elevated in 6 DIV neurons treated with H2O2, suggesting that SIRT1 transcription may be impaired in more mature neurons. Previous studies examining SIRT1 have all used H2O2 as an oxidative stressor and have not explored the effects of alternate stressors such as inhibiting BDNF signaling. Although SIRT1 may still play a role in SUV39H1 activation, it is not the primary driver of H3K9me3 upregulation in neurotrophically deprived neurons.

## Conclusion

In summary, we have found that inhibiting BDNF signaling via TrkB-Fc upregulates H3K9me3, with the greatest elevation occurring in 12 DIV hippocampal neurons. This elevation is at least partially mediated by oxidative stress, as antioxidant treatment prevented H3K9me3 from increasing in TrkB-Fc and H2O2 treated cultures. Although SIRT1 is elevated by H2O2, this treatment does not lead to H3K9me3 upregulation, suggesting that a SIRT1 independent pathway is the primary regulator of hippocampal H3K9me3. Our findings give insight into the epigenetic effects of BDNF on hippocampal neurons.

## Methods

### Hippocampal Neuron Cultures

Primary cultures of dissociated hippocampal neurons were prepared from E17-19 Sprague-Dawley rats by dissection in calcium- and magnesium-free buffer, then digested in 0.125% trypsin at 37°C for 7 min with inversions every minute. The neurons were gently pelleted and re-suspended in 1 ml of growth media, containing neurobasal media with penicillin-streptomycin, B27 (Fisher 17504044), and glutamax. Cells were triturated using three fire-polished pipettes and strained through a 40 μM cell strainer. Cells were plated at 6 × 10^5^ cells/9.5 cm^2^ on plastic plates coated with 0.125 mg/ml poly-d-lysine and maintained at 37°C in a 5% CO_2_ atmosphere in growth media. Cells were fed twice weekly by 50% media exchange. Prior to treatment, 50% of the culture media was replaced twice with antioxidant deprived treatment media consisting of neurobasal media with B27-AO (without antioxidants, Fisher 10889038), penicillin-streptomycin and glutamax. Our methods produce cultures that are over 95% neuronal with few to no glial cells, as established by a previous publication (Tong et al., 2008). Cultures were cultured for 6 DIV to resemble “young” neurons, 12 DIV to resemble “mature” neurons and 21 DIV to resemble “aged” neurons in accordance with a previous publication (Calvo-Rodríguez et al., 2016).

### Culture Treatments

Hippocampal neuron cultures were treated with varying concentrations (10–200 μM) of H2O2 diluted in treatment media for 5 min or 200 μM H2O2 for 4 h. Lyophilized TrkB-Fc (R&D Systems, 688-TK-100) was diluted to a stock solution of 100 μg/ml in sterile PBS. Cultures were treated with 1 μg/ml TrkB-Fc for 24 h. 50% of the culture media was replaced twice with antioxidant deprived media (B27-AO; Fisher 10889038) at the time of treatment with exogenous stressors. For antioxidant treatment, 50% of culture media was replaced twice with growth media containing B27 (Fisher 17504044) at the time of treatment with exogenous stressors.

### Western Blot

Cells were plated at 2.5 × 10^5^ cells/ml in 6 well plates coated with 0.125 mg/ml poly-l-lysine and washed. Media was removed from all wells and ice-cold PBS was used to wash the cells. Cells were lysed in RIPA buffer containing protease inhibitor cocktails from Pierce. Cell homogenates were harvested, sonicated for 6 s and spun at 12,000 × g for 12 min. The supernatant was removed for microBCA assay (Fisher 23235) and western blot analysis. Equal amounts of protein were loaded into 10 or 15% Bio-Rad Tris-HCl gels and transferred onto PVDF membranes using a Bio-Rad Turbo Transfer system. Membranes were washed in tris-buffered saline with 0.1% tween 20 (TBS-T), blocked in 5% bovine serum albumin (BSA) in TBS-T, incubated in primary antibodies overnight in 2.5% BSA in TBS-T at 4°C (anti-H3K9me3, ab8898; anti-beta actin, Cell Signaling Technologies #4967; anti-SIRT1, ab110304; anti-H3, ab1791), washed and incubated in horseradish peroxidase secondary antibodies in TBS-T at room temperature. H3K9me3 was normalized to actin and SIRT1 was normalized to H3. Normalized values are relative to untreated controls. Membranes were developed using Pierce ECL detection kit and immunoreactivity was quantified using ImageJ.

### LDH

Cells were plated at 2.5 × 10^5^ cells/ml in 24 well plates coated with 0.125 mg/ml poly-L-lysine and washed. LDH release was analyzed using a CyQyant LDH assay according to the manufacturer’s instructions (C20301). In brief, 50 μl 10 × lysis buffer was added to maximum LDH control wells and 50 μl ddH20 was added to spontaneous LDH control wells 45 min before the assay. 50 μl of sample medium was transferred from each culture well to a 96 well plate, and samples were measured in duplicate. Media was incubated with 50 μl reaction mixture for 30 min before the addition of 50 μl stop solution. Absorbance was measured at 490 and 680 nm. To determine LDH activity, the 680 nm value was subtracted from the 490 nm absorbance before calculation of percent cytotoxicity [(compound treated LDH activity—spontaneous LDH activity)/(maximum LDH activity-spontaneous LDH activity) *100] and normalization to control conditions (100% survival) to determine relative survival.

### GSH

GSH was quantified using a detection assay kit (ab138881) according to the manufacturer’s instructions. In brief, cells or tissue were washed in PBS and homogenized in 0.5% NP-40 in PBS. Samples were centrifuged for 15 min at 14,000 × g at 4°C and the supernatant was collected. Samples were deproteinized by treatment with trichloroacetic acid (TCA), centrifuged at 12,000 × g for 5 min at 4°C and neutralized with NaHCO3 to a pH of 4–6. Samples were centrifuged at 13,000 × g for 15 min at 4°C and the supernatant was collected. 50 μl of sample was placed in the wells of a black 96 well plate and incubated with 50 μL of GSH assay mixture (1 × thiol green solution) for 1 h. Fluorescence was measured using a microplate reader at an excitation of 490 and an emission of 520 nm.

### Statistical Analysis

The results of ANOVA and post hoc analyses are reported. In all cases, *p* ≤ 0.05 was considered significant. Post hoc multiple comparisons tests are specified in the figure captions. ImageJ was used for image analysis, and Prism was used for data analysis. All graphs represent means ± SEM.

## Data Availability

The original contributions presented in the study are included in the article/Supplementary Material, further inquiries can be directed to the corresponding author.
