# Supplementary material for: Inhibiting BDNF Signaling Upregulates Hippocampal H3K9me3 in a Manner Dependent On In Vitro Aging and Oxidative Stress
Source: Front Aging. 2022 Apr 28;3:796087. doi: 10.3389/fragi.2022.796087 (PMC9261402; doi:10.3389/fragi.2022.796087)
Supplement: Supplementary file 1 [file DataSheet1.pdf]

Supplementary Figures

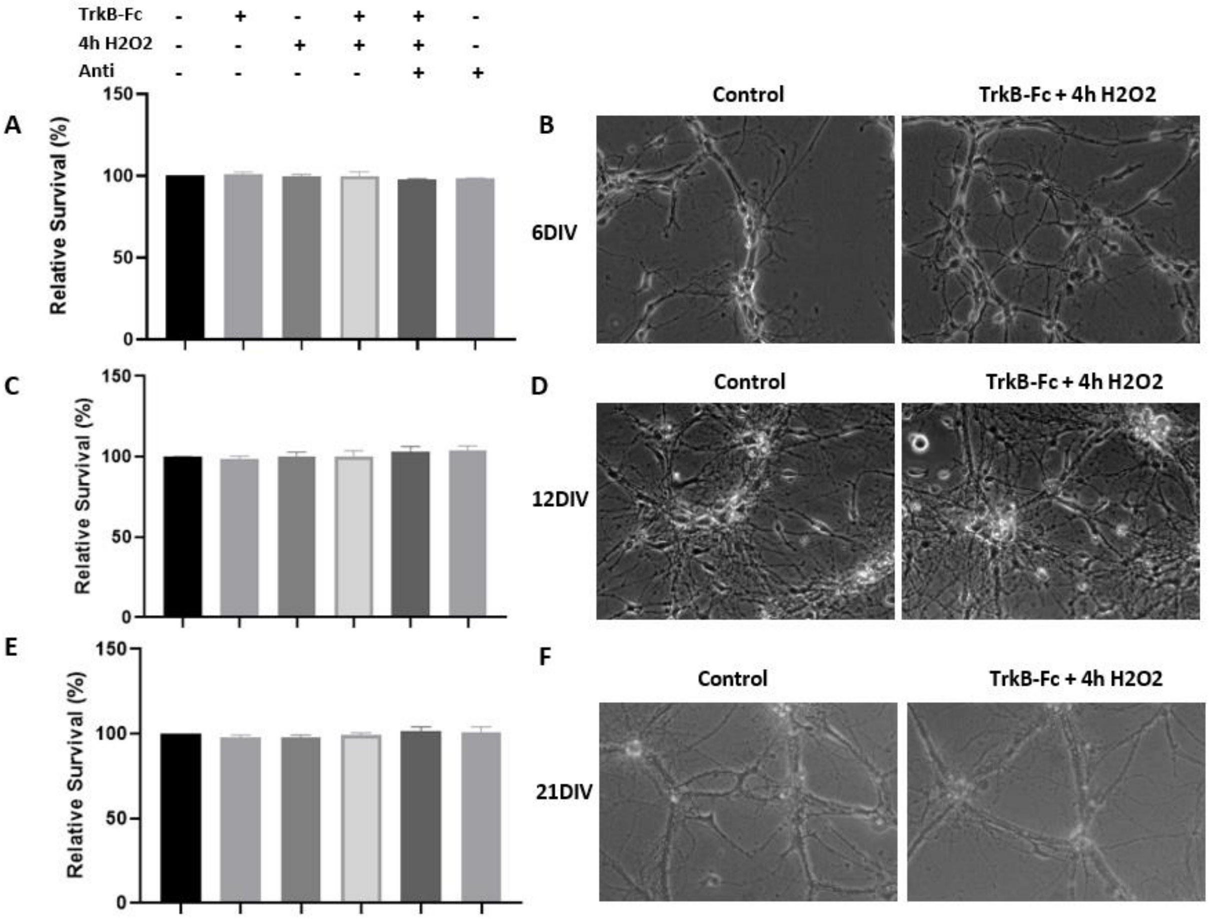

Supplemental Figure 1. TrkB-Fc and H2O2 Do Not Affect Survival in Hippocampal Neuron Cultures. A, LDH survival assay of 6 DIV hippocampal neuron cultures shows no significant change in survival with TrkB-Fc, H2O2 and antioxidants. One way ANOVA, $p=0.36$, $n=4$. B, Representative images of 6 DIV negative control cultures and cultures treated with TrkB-Fc + 4h H2O2. C, LDH survival assay of 12 DIV hippocampal neuron cultures shows no significant change in survival with TrkB-Fc, H2O2, or antioxidants. One way anova, $*p=0.039$, $n=4$. D, Representative images of 12 DIV negative control cultures and cultures treated with TrkB-Fc and 4h H2O2. E, LDH survival assay of 21 DIV hippocampal neurons treated with TrkB-Fc, H2O2 and antioxidants. One way ANOVA, $p=0.714$, $n=4$. F, Representative images of 21 DIV negative control cultures and cultures treated with TrkB-Fc and 4h H2O2.

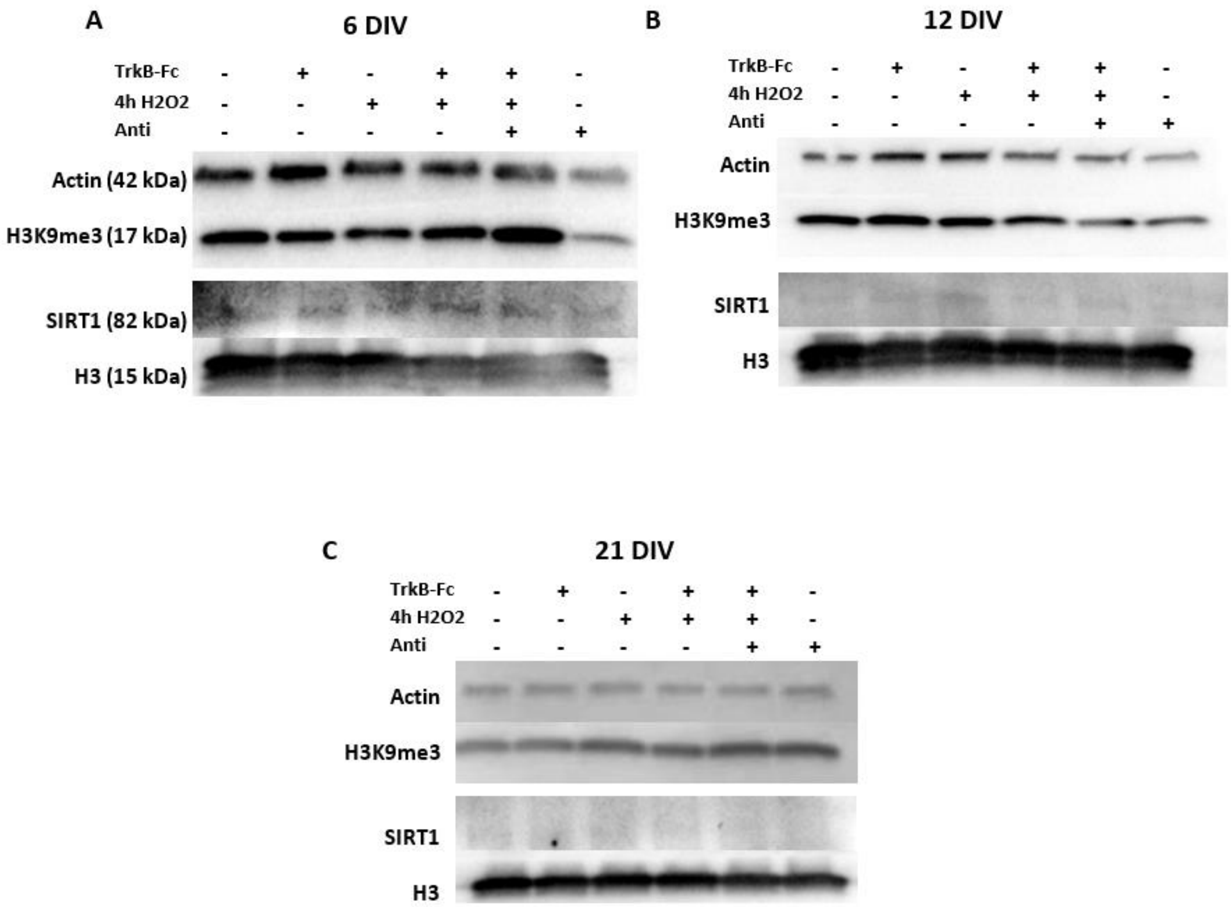

Supplemental Figure 2. Representative Western Blot Images for 6, 12 and 21 DIV Cultures.
Representative western blot images for 6 DIV(A), 12 DIV (B) and 21 DIV neurons (C) stained for SIRT1, actin, and H3. Protein concentration was the same for all wells within a blot, ranging from 8-15 µg/well.

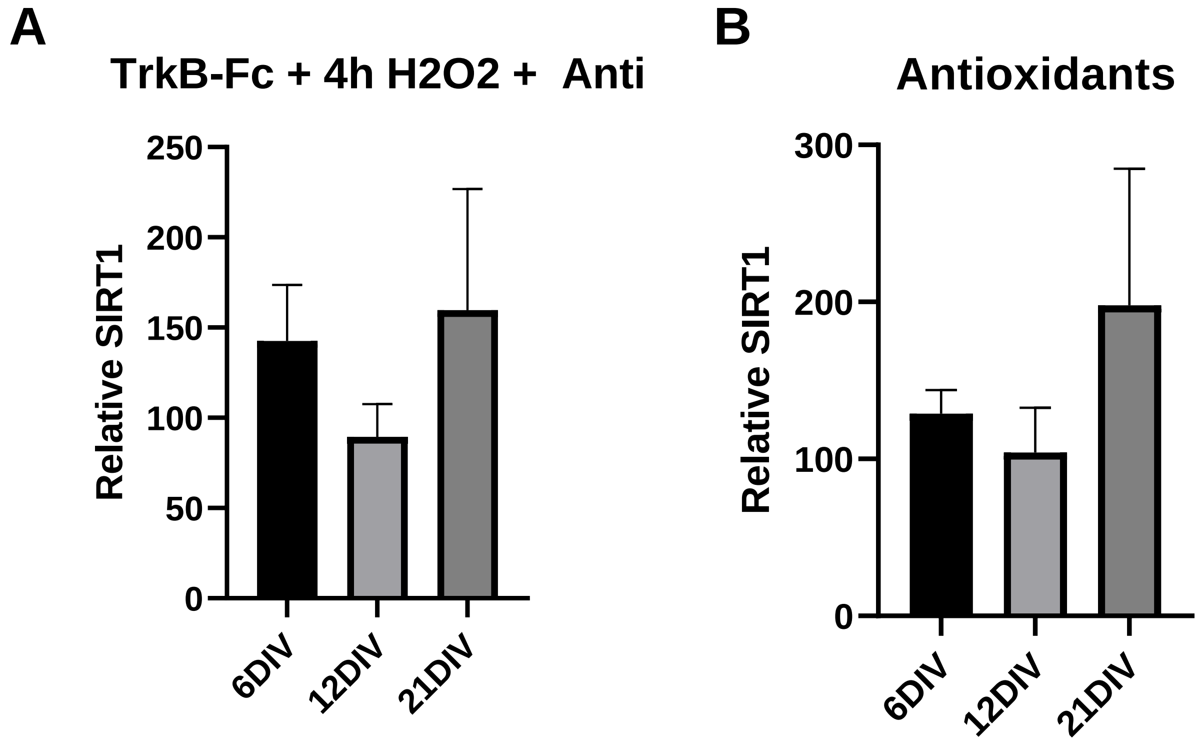

Supplementary Figure 3. Antioxidant Treatment Does Not Change SIRT1 Levels. A, SIRT1 western blot analysis of hippocampal neurons treated with 1 µg/ml TrkB-Fc for 24h, 200 µM H_2O_2 for 4h and antioxidants for 24h. Welch's ANOVA, $p=0.38$. B, SIRT1 western blot analysis of hippocampal neurons treated with antioxidants for 24h. Welch's ANOVA, $p=0.55$.
